# A-Kinase Anchoring Protein 79/150 Scaffolds Transient Receptor Potential A 1 Phosphorylation and Sensitization by Metabotropic Glutamate Receptor Activation

**DOI:** 10.1038/s41598-017-01999-4

**Published:** 2017-05-12

**Authors:** Allison Doyle Brackley, Ruben Gomez, Kristi A. Guerrero, Armen N. Akopian, Marc J. Glucksman, Junhui Du, Susan M. Carlton, Nathaniel A. Jeske

**Affiliations:** 1 Departments of Pharmacology, University of Texas Health Science Center at San Antonio, Texas, 78229 USA; 2 Oral and Maxillofacial Surgery, University of Texas Health Science Center at San Antonio, Texas, 78229 USA; 3 Endodontics, University of Texas Health Science Center at San Antonio, Texas, 78229 USA; 4 Physiology, University of Texas Health Science Center at San Antonio, Texas, 78229 USA; 5 Midwest Proteome Center and Department of Biochemistry and Molecular Biology, Chicago Medical School, Rosalind Franklin University of Medicine and Science, North Chicago, IL 60064 USA; 6 Department of Neuroscience and Cell Biology, University of Texas Medical Branch, Galveston, TX 77555 USA

## Abstract

Mechanical pain serves as a base clinical symptom for many of the world’s most debilitating syndromes. Ion channels expressed by peripheral sensory neurons largely contribute to mechanical hypersensitivity. Transient Receptor Potential A 1 (TRPA1) is a ligand-gated ion channel that contributes to inflammatory mechanical hypersensitivity, yet little is known as to the post-translational mechanism behind its somatosensitization. Here, we utilize biochemical, electrophysiological, and behavioral measures to demonstrate that metabotropic glutamate receptor-induced sensitization of TRPA1 nociceptors stimulates targeted modification of the receptor. Type 1 mGluR5 activation increases TRPA1 receptor agonist sensitivity in an AKA-dependent manner. As a scaffolding protein for Protein Kinases A and C (PKA and PKC, respectively), AKAP facilitates phosphorylation and sensitization of TRPA1 in *ex vivo* sensory neuronal preparations. Furthermore, hyperalgesic priming of mechanical hypersensitivity requires both TRPA1 and AKAP. Collectively, these results identify a novel AKAP-mediated biochemical mechanism that increases TRPA1 sensitivity in peripheral sensory neurons, and likely contributes to persistent mechanical hypersensitivity.

## Introduction

A-kinase Anchoring Protein 79/150 (AKAP) is a scaffolding protein expressed throughout neural tissues^1^. Importantly, AKAP facilitates post-translational modifications of plasma membrane receptors by kinases including Protein Kinase A (PKA) and Protein Kinase C (PKC^2^). AKAP is required for PKA phosphorylation and sensitization of the Transient Receptor Potential Vanilloid 1 (TRPV1) receptor in sensory neurons following inflammatory insult^3–5^. Moreover, G-Protein Coupled Receptor (GPCR)-mediated Phospholipase C (PLC) activation increases AKAP association with TRPV1^6^. Inflammatory mediators, including those that activate metabotropic bradykinin and glutamate receptors, activate downstream PLC-, PKC- and PKA-pathways^7–9^. Therefore, peripheral inflammatory insult stimulates AKAP-dependent post-translational signaling mechanisms that reduce ligand-gated ion channel thresholds and increase peripheral somatosensitivity in both acute and persistent pain behavioral measures. These studies identify AKAP as an important neuronal scaffolding mediator that facilitates inflammatory somatosensitization of peripheral tissues to stimuli.

Glutamate serves as the primary excitatory neurotransmitter in the nervous system. Studies have identified that glutamate is released in the periphery following neuronal depolarization^10^, and sensitizes peripheral sensory neurons through Type 1 metabotropic glutamate receptors (mGluRs) including mGluR5^11, 12^. mGluR5 is a GPCR primarily coupled to Gαq/11, which signals downstream to activate PLC^7, 13^ and PKA^8, 14, 15^. Importantly, both PLC and PKA are implicated in inflammatory signaling mechanisms in sensory neurons, and modify ion channels to induce behavioral states of hyperalgesia and allodynia^3, 9, 16–18^. Indeed, previous work has identified a functional role for mGluR5 in inflammatory mechanical hyperalgesia^19^, which can grow into a persistent state, thereby reducing patient quality of life. Few studies have identified mechanisms by which inflammation can precipitate a state of persistent mechanical hypersensitivity. Furthermore, few studies have identified metabotropic and/or ionotropic receptors that contribute to persistent mechanical hypersensitivity.

Transient receptor potential family A1 (TRPA1) is an important ligand-gated ion channel involved in acute and inflammatory pain^20–22^. TRPA1 is activated acutely by chemical and physical agonists including electrophilic agonists allyl isothiocyanate (AITC, Mustard Oil/MO) and cinnamaldehyde, and mechanical stimuli^22–25^. This activation increases the calcium permeability of the receptor, priming membrane depolarization and subsequent sensory neuron activation^26^. Previous work has identified mechanisms that contribute to increased TRPA1 responses, including increased plasma membrane expression and intracellular di-sulfide covalent bonding^23, 27^, yet no one has identified a reversible post-translational mechanism that sensitizes the receptor to persistent stimulus-evoked activation. Experimental observations presented herein demonstrate that an AKAP-dependent mechanism post-translationally modifies TRPA1 to contribute to persistent mechanical hypersensitivity.

In this study, experimental results indicate that AKAP scaffolds PKA and PKC following inflammatory insult to post-translationally modify TRPA1. Data presented here demonstrate that mGluR5 activation in sensory neurons persistently sensitizes TRPA1 to mechanical activation. Therefore, targeting AKAP and/or TRPA1 could reduce persistent mechanical hypersensitivity to improve inflammatory phenotypes for millions of patients.

## Materials and Methods

### Reagents

(*S*)-3,5-Dihydroxyphenylglycine (DHPG), 3-((2-Methyl-1,3-thiazol-4-yl)ethynyl) pyridine hydrochloride (MTEP), and fenobam were purchased from Tocris/R&D Systems (Minneapolis, MN). Mustard oil (MO) and all other chemicals were purchased from Sigma Aldrich, unless otherwise noted.

### Animals

Procedures using animals were approved by the Institutional Animal Care and Use Committees of The University of Texas Health Science Center at San Antonio (UTHSCSA) and The University of Texas Medical Branch at Galveston (UTMB). Moreover, these procedures were conducted in accordance with policies for the ethical treatment of animals established by the National Institutes of Health and International Association for the Study of Pain.

### Tissue Culturing

Male Sprague-Dawley rats (175–200 g, Charles River Laboratories, Wilmington, MA) and AKAP wild-type (WT)/knockout (KO) mice with a C57/Bl6 background (4–10 weeks of age) were used. Trigeminal ganglia (TG) were removed bilaterally from male rodents, and dissociated by collagenase treatment (30 min, Worthington, Lakewood, NJ), followed by trypsin treatment (15 min, Sigma, St.Louis, MO). Cells were centrifuged and re-suspended between each treatment with Pasteur pipettes. Rodent dorsal root ganglia (DRG, L4-6) were bilaterally dissected and dissociated by collagenase and dispase (40 min) co-treatment. TG or DRG were then centrifuged, aspirated, and re-suspended in Dulbecco’s Modified Eagle Medium (DMEM, Gibco, Grand Island, NY) with 10% fetal bovine serum (FBS, Gibco), 250 ng/ml nerve growth factor (NGF, Harlan, Indianapolis, IN), 1% 5-fluoro deoxyuridine (Sigma), 1% penicillin/streptomycin (Gibco), and 1% L-glutamine, and then placed on plates coated with poly-D lysine, or coverslips coated with poly-D lysine and laminin (Corning, Corning, NY). Cultures were maintained at 37 °C, 5% CO_2_, and grown in 10 cm plates for 5–7 days for biochemistry experiments. Cultures for electrophysiology and calcium imaging were grown 18–36 h. AKAP150 WT and KO mice were originally created and characterized in the laboratory of John D. Scott^28^, and maintained at UTHSCSA. All WT and KO animals used for experiments were littermates.

### Cell Use

Chinese Hamster Ovary (CHO) cells were utilized for transient transfection and expression of specified AKAP and TRPA1 constructs, following previous published protocol^29^. CHO cells were utilized from divisions 7–20, and were transfected with Lipofectamine 2000, following manufacturer’s instructions (Thermo Fisher Scientific, Waltham, MA). All cells were transfected with indicated cDNA constructs along with empty Green Fluorescent Protein (GFP) vector to identify positively-transfected cells. Cells were analyzed 24 h following transient transfection.

### TRPA1 Antibody Verification

Trigeminal Ganglia were dissected bilaterally and cultured from six rats onto 2 10-cm plates, and grown for 5 days, as outlined above. Cultures were lysed, and cleared cell lysates were incubated with TRPA1 antibody (Neuromics, Edina, MN^30^) for and precipitated as outlined below. Samples were resolved by SDS-PAGE, and acrylamide gel was excised from the 120 kDa region, corresponding to rat TRPA1, and processed for mass spectrometric analysis.

### In-gel digestion of proteins

The excised protein gel band was cut to small particles with a spot picker and washed twice with HPLC-grade water (Fisher) and incubated with 1:1 v/v of 0.1 NH_4_HCO_3_ (Sigma) for 15 min. Wash solution was removed and HPLC-grade acetonitrile (Fisher) was then added to cover the gel particles for 1 hr, acetonitrile removed and gel particles were rehydrated in 0.1 M NH_4_HCO_3_ for 10 min. An equal volume of ACN was then added for a 1:1 v/v of 0.1 NH_4_HCO_3_/ACN. After 10 min all liquid was removed and the gel particles dried under vacuum then reduced with 10 mM dithiothreitol (DTT, Thermo Scientific) and alkylated with 55 mM iodoacetamide (GE Healthcare) in 0.1 M NH4HCO3. After reduction and alkylation, gel particles were washed and trypsin digestion for 24 h at 37 °C. Resulting peptides were extracted by addition of a 25 mM NH4HCO3, 5% formic acid and acetonitrile (2:1:1). The tryptic digest mixture was desalted with a C18 ZipTip (Millipore) and eluted into 60% methanol containing 0.1% formic acid. Pooled digested peptides were dried and re-suspended in formic acid: water: acetonitrile (0.1:95:5) for liquid chromatographic separation and electrospray ionization mass spectrometry (LC-ESI MS) analysis.

### Identification of proteins with LC-MS/MS

The re-suspended tryptic digest was first separated on a reversed-phase column (C-18 Acclaim PepMap100, 75 um × 15 cm, Thermo Scientific). Specifically, solvent A was 0.1% formic acid in 5% ACN and solvent B was 0.1% formic acid in 95% ACN. The peptides were first loaded onto a pre-column (u-Precolumn, 5 mm, 30 um i.d. × 10 cm, Thermo Scientific) using solvent A for 5 min and then peptides were eluted through the analytical column using a long gradient at a flow rate of 300 nL/min.

Eluting peptides were then electrically sprayed into a LTQ Orbitrap Elite mass spectrometer (Thermo Scientific) with 2000 V electrospray voltage applied on the ESI emitter tip. The MS was operated in data-dependent top 10 most intense ion mode with the following settings: (1) Mass range setting: 200–1800 Da; (2) Resolution for FT precursor scan: 120,000; (3) Lock mass: 445.120025 Da; (4) AGC target: 3E6 for FT, 1E4 for IT; (5) Max injection time: 50 ms; (6) FT scan: precursor; (7) Collision mode: CID with ion trap product scan. The scanned ions were assigned the charge state in the range of +2 to +4 with optimized isolation width and dynamic exclusion.

Protein identifications were performed with PEAKS (v. 8.0, Bioinformatics Solutions Inc.) with the following settings: trypsin was specified with one non-specific cleavage for the database search. Carbamidomethylation (mono mass 57.0215 Da) was set on cysteine residues as fixed modification and oxidation (mono mass 15.9949 Da) on methionine residues as variable modification. The mass error tolerance for precursor ions was 10 ppm and 0.5 Da for fragment ions. Filter quality was set >0.65. MS scan mode was set as FT/Orbitrap and MS/MS scan mode set as linear ion trap with fragmentation mode CID. False discovery rate (FDR) was set to 1% on peptide and protein levels with defaults for dynamic score refinement, precursor scoring, interference correction, normalization, and Q value.

In Supplementary Figure 1, 13 peptides unique to rat TRPA1 were identified by MS analysis, demonstrating specificity of the Neuromics antibody used for immunoprecipitation and Western blot identification.

### Co-immunoprecipitation, Orthophsphate Labeling and Western Blotting

For co-immunoprecipitation, TG cultures in 10-cm plates were treated and prepared for homogenization and isolation of crude plasma membrane fractions, as described previously^3^. Crude plasma membrane homogenates are quantified for protein concentration by Bradford analysis^31^. Equal samples underwent co-immunoprecipitation (100 μg total protein) or gross homogenate WB analysis (10 μg total protein). Samples isolated for co-immunoprecipitation were diluted to 500 μl with homogenization buffer^3^ and incubated with antibodies specific to AKAP150 (1 μg, Santa Cruz Biotechnology, Santa Cruz, CA^3^), pulled down with protein agarose-A (SigmaAldrich), and resolved by Sodium Dodecyl Sulfate -Polyacrylamide Gel Electrophoresis (SDS-PAGE). Gels were transferred to polyvinyldifluoride membranes (PVDF, EMD Millipore, Billerica, MA), blocked in 5% non-fat milk in Tris buffered saline with 0.1% Tween-20 non-ionic detergent, and incubated 18 h with primary antibody anti-TRPA1 (Neuromics) or anti-AKAP150. Anti-rabbit or –mouse secondary antibodies (GE Healthcare Life Sciences, Piscataway, NJ) were applied to rinsed blots, incubated at RT for 1 h, and then blots were rinsed again. Blots were incubated with enhanced chemiluminescence solution (GE Healthcare Life Sciences), exposed to X-ray film and developed for analysis.

For phosphorylation experiments, TG cultures in 10-cm plates were incubated with 0.5 mCi^32^P-orthophosphate (Perkin Elmer, Waltham, MA) per plate for 4 hr at 37 °C, treated as indicated for the specified times, and harvested as originally described^3^. Equal volumes of cell lysate were prepared for immunoprecipitation (90% of total lysate, using anti-TRPA1 (Neuromics) and lysate protein expression (10% of total lysate), and processed as above for SDS-PAGE. Samples were transferred to PVDF membrane, and incubated with autoradiography film overnight at −80 °C, prior to development and eventual Western blotting (WB) for total TRPA1.

All films were scanned and immunoreactive bands were quantified using Image J shareware.

### Calcium Imaging

DRG neurons and CHO cells were incubated for 30 min at 37°, 5% CO_2_, with Fura2-AM (2 µM, Invitrogen Life Technologies, Grand Island, NY) in the presence of 0.05% Pluronic (Invitrogen Life Technologies) for 30 min or 1 h, respectively. Experiments were performed with an inverted Nikon Eclipse TE-2000-U microscope, equipped with 20X/0.75 numerical aperture Fluor objective. Cultured cells were excited at the 340 and 380 nm wavelength from a Lambda LS Fluorescent light source (Sutter Instruments), while a Hamatasu digital CCD Camera detected emissions at 510 nm. Collected data were analyzed with MetaFluor Software (Molecular Devices). Ratiometric 340/380 nm values were converted into nM of Ca^2+^ according to the formula [Ca^2+^] = β(R − R_min_)/(R_max_ − R); where β = 380 nm value in Ca^2+^-free/Ca^2+^-saturating solution and R = 340/380 ratio and all the other values are specific to the experimental setup and were determined with use of a Fura-2 AM calibration kit (Invitrogen Life Technologies).

Experiments were performed at room temperature (RT) with use of a PM2000 Microinjector (MicroData Instruments, Inc.), multichannel perfusion valve controller (Warner Instrument Corporation), and multi-barrel glass pipette, which allowed for continuous exchange of bath solution during drug application. The micropipette, which delivered drug-containing solutions, was precisely placed in the optic field that includes our cells of interests. Both bath and pipet solution were under an automatically controlled pressurized system that, in pairing with pneumatic pump, supported precise, fast and consistent fluid flow and exchange. Recordings were performed in standard extracellular solution (SES) that contained the following (in mM): 140 NaCl, 5 KCl, 2 MgCl_2_, 2CaCl_2_, 10 HEPES, 10 Glucose, pH 7.4 [NaOH], which also served as a vehicle for all drugs used for Ca^2+^ imaging.

Coverslips containing transfected CHO cells were pre-treated for 5 min with 8-Br-cAMP (PKA activator, 10 μM) or Phorbol 12,13-dibutyrate (PDBu, PKC activator, 1 μM), during which time corresponding filters were used to select GFP-positive cells. As SES was bath perfused, 60 sec basal Ca^2+^ levels were recorded for each cell, followed by 30 sec microinjection of mustard oil (MO, 50 μM) under continuous SES bath perfusion. Cell recordings continued for an additional 270 sec after MO exposure concluded. Similarly, coverslips of nucleofected DRG neurons were pre-treated for 5 min with 8-Br-cAMP (10 μM) or DHPG (10 μM, Tocris^32^) before recordings began and a corresponding filter was used to select GFP-positive neurons. As with CHO cells, DRG neuron basal Ca^2+^ levels were recorded prior MO (50 μM, 30 sec) exposure via pipet application; recordings continued an additional 270 s. However, DRG neurons were additionally subjected to capsaicin (1 μM, Sigma Aldrich) treatment at the end of the experiment, as CAP-sensitivity of recorded cells indicates positive sensory neuronal phenotype within the heterogeneous culture. Data were analyzed by an unpaired student’s *t* test for each cDNA tested.

### Site-directed Mutagenesis

Myc-labeled mouse TRPA1 cDNA was gifted by Ardem Patapoutian (Scripps Research Institute, CA). Polymerase chain reaction (PCR) was employed using a Quickchange II XL Site-directed Mutagenesis kit, following directions from the manufacturer (Agilent Technologies, Santa Clara, CA). Primers were custom designed and created by Integrate DNA Technologies (Coralville, IA), and respectively correspond to the following mutants, in 5′-3′ directionality: S87A Forward, CTGATCATCAATGGTTCTGCGTGTGAAGTGCTG, Reverse, CAGCACTTCACACGCAGAACCATTGATGATCAG; S119G Forward, GTGTAAAGTTTCTTCTCGGCCAAGGAGCAAATCCAAAC, Reverse, GTTTGGATTTGCTCCTTGGCCGAGAAGAAACTTTACAC; S179G Forward, CGTGTGCCAAAGACAACGGTGAAGCTTTGCAAATTTTG, Reverse, CAAAATTTGCAAAGCTTCACCGTTGTCTTTGGCACACG; T281A Forward, CTCCATTTTGCTGCAGCCCAGGGAGCCACTG, Reverse, CAGTGGCTCCCTGGGCTGCAGCAAAATGGAG; S318A Forward, CTGCTTCACAGAGCCGCGTTATTTGATCACCATG, Reverse, CATGGTGATCAAATAACGCGGCTCTGTGAAGCAG; S441A Forward, GGCTTCAATGTGTCCATTCATGGCAAAAGTAAAGATAAGAAGTCG, Reverse, CGACTTCTTATCTTTACTTTTGCCATGAATGGACACATTGAAGCC; S445G Forward, CTGCATTTTGCAGCCGGTTATGGGCGCATC, Reverse, GATGCGCCCATAACCGGGTGCAAAATGCAG; T529A Forward, GGTGGGTACACTCAGGCCATGAAGGTCATTC, Reverse, GAATGACCTTCATGGCCTGAGTGTACCCACC; T536A Forward, CCATGAAGGTCATTCTTGATGCTAACTTGAAATGCACAGAC, Reverse, GTCTGTGCATTTCAAGTTAGCATCAAGAATGACCTTCATGG; S1101G Forward, GGCTGGAACAGATGCACGGCAAGTGGAATTTTGTC, Reverse, GACAAAATTCCACTTGCCGTGCATCTGTTCCAGCC. All mutations were confirmed by sequencing by the UTHSCSA Nucleic Acids Core Facility.

### Electrophysiological Recordings: *In Vitro* Skin-Nerve Preparation

The preparation used to record from nociceptors has been previously described in detail^33, 34^. Briefly, male AKAP WT and KO mice (4–10 weeks of age) were sacrificed with an overdose of CO_2_. The glabrous skin was dissected from each hindpaw starting at the ankle and moving to the tips of the toes. The medial and lateral plantar nerves were also dissected free and kept intact with the glabrous skin. The preparation was placed corium side up in a 2-chambered tissue bath. The nerves were drawn into one chamber and the skin laid out and pinned to the gel floor of the other chamber.

In the recording chamber the nerves were de-sheathed and carefully teased apart so that small nerve bundles could be obtained. These were laid across the gold wire-recording electrode. A glass rod was pressed into the glabrous skin to find receptive fields of units in the nerve bundles. Once the receptive field of a unit was located, the conduction velocity (CV) was measured and a sequence of testing was performed as described below.

### Mechanical and Chemical Testing Procedures

For mechanical stimulation, a Dual-Mode Lever System (Aurora Scientific Inc. Ontario, Canada) was used to determine the mechanical threshold and discharge rate of each unit studied. A motor-driven stylus (0.7 mm diameter) was placed in the most sensitive place in the unit receptive field on the corium side and delivered a force in the form of a continuous ascending ramp from 0–250 mN over 20 sec. A unit was considered mechanically sensitive if the discharge rate evoked during stimulation was greater than the background mean frequency for that fiber during the 20 sec immediately preceding the mechanical stimulation. When the first spike occurred after activation of the ramp, the force was noted and this was considered threshold for that unit. A suprathreshold square wave pulse (250 mN, 10 sec) was also applied. The discharge rate during this stimulus was reported as the mechanical discharge rate for that unit.

To investigate unit responses to chemicals, a small plastic ring (4.5 mm diameter) was placed over the receptive field of each unit. The synthetic interstitial fluid (SIF) in the ring was replaced with a drug dissolved in SIF (pH 7.40). As with any *in vivo* intraplantar injections, the drugs applied in this *in vitro* set-up must diffuse through the plantar connective tissue, dermis and epidermis to reach the nociceptor nerve terminals, thus drug concentrations are considerably diluted by the time they reach their target sites.

To determine the percent of units responding to a drug application, only those units whose discharge rate increased more than the mean +2 standard deviations (calculated using the background discharge rate for the population) were considered “responders”.

### Behavior

The transition from acute to chronic pain involves multiple variables, including mechanisms of post-translational modification in peripheral sensory neurons. To study this phenomenon, the Levine research group characterized a behavioral model of hyperalgesic priming to study biochemical and molecular contributors to the transition from acute to chronic pain^35, 36^. As previously documented, this behavioral model follows a protocol by which peripheral injection of carrageenan several days prior to a subsequent inflammatory event results in a significant temporal extension of somatosensitivity. This behavioral protocol was recently employed to study the role of mGluR5 in persistent thermal sensitivity^37^, demonstrating an important role for AKAP79/150 (AKAP) scaffolding of TRPV1. Furthermore, this work established that a local injection protocol into the hind paw failed to elicit systemic effects on the contralateral side, as demonstrated previously^35, 36^. Past studies demonstrate a peripheral role for mGluR5 in *in vivo* models of pain behavior^11^. Here, we investigated the role of mGluR5 in persistent mechanical hypersensitivity in concert with AKAP, using the same approach.

Mechanical withdrawal threshold of the rodent paw was measured with a plantar electronic von Frey Anesthesiometer (ALMEMO 2450 Ahlborn, IITC, Woodland Hills, CA). Briefly, animals were placed in plastic boxes with a mesh floor surface for a 30 min habituation period. For rats and mice, the “rigid tip” was used with a 0.8mm uniform tip diameter. The amount of force generated that elicited hindpaw withdrawal was automatically measured and recorded as paw withdrawal threshold in grams (g). Measurements were taken in triplicate at least 30 sec apart and the average was used for statistical analysis. Data are presented as mean paw withdrawal threshold ± SEM paw withdrawal thresholds. Observers were blinded to the treatment conditions and genotypes of the animals.

All injections were given intraplantarly (*ipl*) in 50 μl (rat) or 10 μl (mouse) volumes via a 28-gauge needle inserted through the lateral footpad just under the skin to minimize tissue damage. Prostaglandin E2 (PGE_2_) drug doses were taken from previous work by Aley *et al*.^38^. MTEP drug doses were calculated from previous work that identified an ED50 of 2.3 mg/kg for radioligand binding^39^. Drug stocks were dissolved in Dulbecco’s Phosphate Buffered Saline (PBS, Gibco by Thermo Fisher Scientific, Inc.). For rat priming experiments, rats were tested for baseline responses prior to injections, and then injected with carrageenan (50 μl of a 0.1% solution in PBS) or vehicle (PBS). For the next four days, rats were injected with MTEP (400 μg) or vehicle (PBS). Rats were tested again on 5 days post-carageenan for baseline responses, and then injected with PGE_2_ (100 ng) followed by repeated paw withdrawal threshold testing at 0.5, 4, and 24 h post-PGE_2_ injection. For mouse priming experiments, littermate AKAP150 WT and KO littermates, and TRPA1 WT and KO littermates were tested for baseline responses prior to injections, and then injected with carrageenan (10 μl of a 0.5% solution). Mechanical paw withdrawal threshold was measured again 5 days post-carageenan prior to injection with PGE_2_ (100 ng) and repeated mechanical testing at 0.5, 1, 2, and 4 h post-PGE_2_ injection from a minimum of 6 animals.

Nociceptive behavior measurements were also taken from TRPA1 WT and KO littermate mice for 5 min following vehicle (ddH_2_O, 5 μl) or 8-Br-cAMP (10 μM, 5 μl) pre-injection (*ipl)*, followed by mustard oil (MO, 10 μl of 0.01% solution) injection (*ipl)*. Nociceptive behavior including licking, biting, scratching, and/or petting the injected area was quantified in seconds over a 5 min period following MO injection. Observers were blinded to the treatment conditions and genotypes of the animals.

### Electrophysiology (Patch-Clamp Recordings)

Dorsal root ganglia (DRG) neurons were dissected bilaterally and cultured for electrophysiology. All recordings were made in whole-cell voltage clamp (holding potential (V_h_) of –60 mV) configuration at 22–24 °C from the somata of sensory neurons (15–40 pF). Data were acquired and analyzed using Axopatch 200B amplifiers and pCLAMP9.2 software (Molecular Devises, CA). Recording data were filtered at 0.5 kHz and sampled at 2 kHz. Borosilicate pipettes (Sutter, Novato, CA) were polished to resistances of 3–5 M□ in the standard pipette solution. Access resistance (R_s_) was not compensated. Data were rejected when R_s_ changed >20% during recording, leak currents were >100 pA, or input resistance was <200 M. Currents were considered positive when their amplitudes were 5-fold larger than displayed noise (in root mean square). Standard external solution (SES) contained (in mM): 140 NaCl, 5 KCl, 2 CaCl_2_, 1 MgCl_2_, 10 D-glucose and 10 4-(2-hydroxyethyl)-1-piperazineethanesulfonic acid (HEPES), pH 7.4. The pipette solution for the perforated patch configurations consisted of (in mM): 140 KCl, 5 NaCl, 10 EGTA, 1CaCl_2_, 1 MgCl_2_, and 10 HEPES pH 7.3. Drugs were applied using a fast, pressure-driven and computer controlled 8-channel system (AutoMate Scientific, San Francisco, CA).

## Results

**Figure 1:**
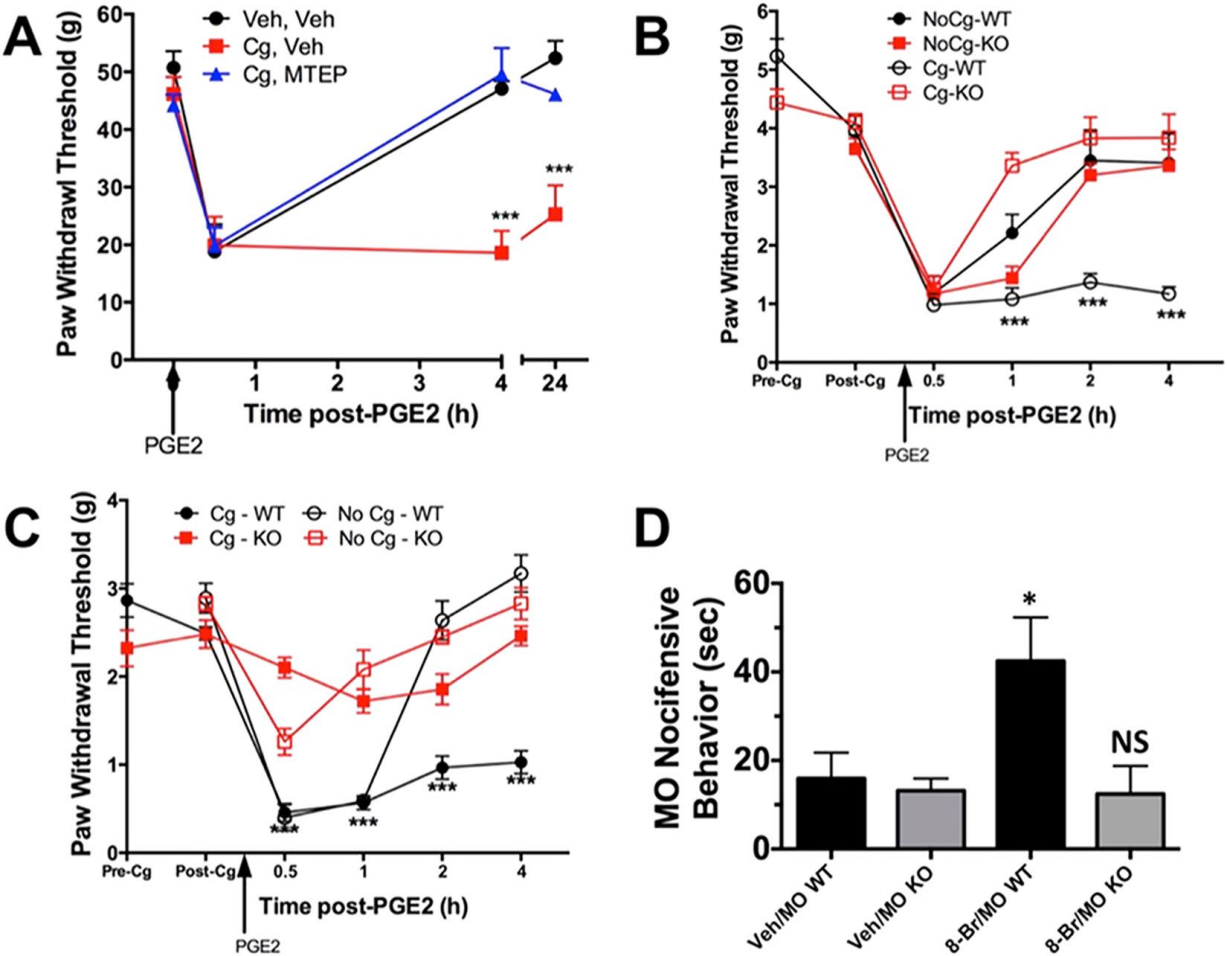
mGluR5, AKAP and TRPA1 Contribute to Mechanical Hyperalgesic Priming Behavior. (**A**) Male Sprague-Dawley rats were injected intraplantarly (*ipl*) with vehicle (Veh) or carrageenan (Cg, 5 μl of 1% solution in 50 μl PBS final). Over the next 4 days, rats were then injected with Veh or MTEP (400 μg in 50 μl PBS, *ipl*). Rats were tested for paw withdrawal threshold (PWT, g) on day 5, then injected with PGE_2_ (100ng in 50 μl PBS, *ipl*), and again tested for PWT 0.5, 4 and 24 h post-PGE_2_. n = 6 rats/treatment paradigm; ***p < 0.005; two-way ANOVA with Bonferroni post-hoc analysis. (**B**) AKAP WT and KO mice (WT and KO, respectively) were tested for PWT (g) before *ipl* injections (Pre-Cg). Mice were then injected with vehicle (No Cg) or Cg (5 μl of 1% solution in 10 μl PBS, final, *ipl*) on Day 1. Mice were tested for baseline PWT on Day 5 (Post-Cg), then injected with PGE_2_ (50ng in 10 μl, *ipl*) and tested for PWT at 0.5, 1, 2, and 4 h post-PGE_2_. n = 6–8 mice/genotype/treatment paradigm, ***p < 0.005; two-way ANOVA with Bonferroni post-hoc analysis. (**C**) TRPA1 WT and KO mice (WT and KO, respectively) were tested and treated identically as in panel B. n = 6 mice/genotype/treatment paradigm; ***p < 0.005; two-way ANOVA with Bonferroni post-hoc analysis. (**D**) TRPA1 WT and KO mice (WT and KO, respectively) were pre-injected with vehicle (Veh, ddH_2_O, 5 μl) or 8-Br-cAMP (8-Br, 10 μM, 5 μl) for 5 min prior to mustard oil (MO, 10 μl of 0.01% solution) injection *ipl*. Nociceptive behavior including licking, biting, scratching, and/or petting the injected area was quantified in seconds over a 3 × 5 min bin collecting period following MO injection. N = 6 mice/genotype/treatment paradigm; *p < 0.05; two-way ANOVA with Bonferroni post-hoc analysis.

Mechanical hyperalgesia and allodynia are commonly reported symptoms of chronic pain. To quantify this behavior in rodents, we employed an electronic von Frey Anesthesiometer to measure paw withdrawal thresholds^40, 41^. Rats pre-injected with carrageenan in the hindpaw experienced persistent mechanical hypersensitivity for 24 h following PGE_2_ administration (Fig. 1A) over rats pre-injected with vehicle. However, antagonism of mGluR5 with MTEP reversed the carrageenan priming effect on PGE_2_-induced mechanical hypersensitivity, indicating an important role for mGluR5 in persistent mechanical pain. To test whether AKAP facilitates peripheral somatosensitization, we extended this work and measured carrageenan priming in AKAP WT and KO. AKAP WT mice pre-treated with carrageenan displayed persistent mechanical hypersensitivity similar to rats (Fig. 1B). However, AKAP KO mice failed to develop persistent mechanical hypersensitivity to PGE_2_ following peripheral exposure to carrageenan.

Several research groups have reported on the role of TRPA1 in inflammatory mechanical hypersensitivity^22, 24, 42–44^. We extended those observations by treating TRPA1 WT and KO mice within the same hyperalgesic priming protocol established in Fig. 1B. Results indicate that TRPA1 expression is required to establish persistent mechanical hypersensitivity to an acute PGE2 inflammatory insult following carrageenan priming (Fig. 1C). Interestingly, mechanical sensitivity was not significantly different between TRPA1 WT and KO mice, indicating that although TRPA1 may contribute to persistent inflammatory mechanical allodynia, it does not serve as the sole transducer in mechanical sensitivity, and is in agreement with previous studies^45, 46^.

Given that PKA plays an important role in hyperalgesic priming^36^, we hypothesized that the PKA scaffolding protein AKAP may play a role in TRPA1 nociceptive behavior. We treated AKAP WT and KO mice with mustard oil (MO, 10 μl of 0.01% solution), a specific chemical agonist of TRPA1^26^, following a pre-injection with vehicle or PKA-activator 8-Br-cAMP (10 μl of 1 mM, 10 min prior to MO), to determine whether PKA can sensitize TRPA1-induced nocifensive behavior in an AKAP-dependent manner. Importantly, measurements over a 15 min period (3 × 5 min bins) immediately following MO injection revealed a significant sensitizing response of 8-Br-cAMP on MO-evoked nocifensive behavior (Fig. 1D, *p < 0.05, two-way ANOVA with Bonferroni correction). However, the PKA-sensitizing effect was absent in AKAP KO animals. Taken together, these studies indicate that AKAP drives the inflammatory sensitization of TRPA1 that contributes to persistent mechanical hypersensitivity.

**Figure 2:**
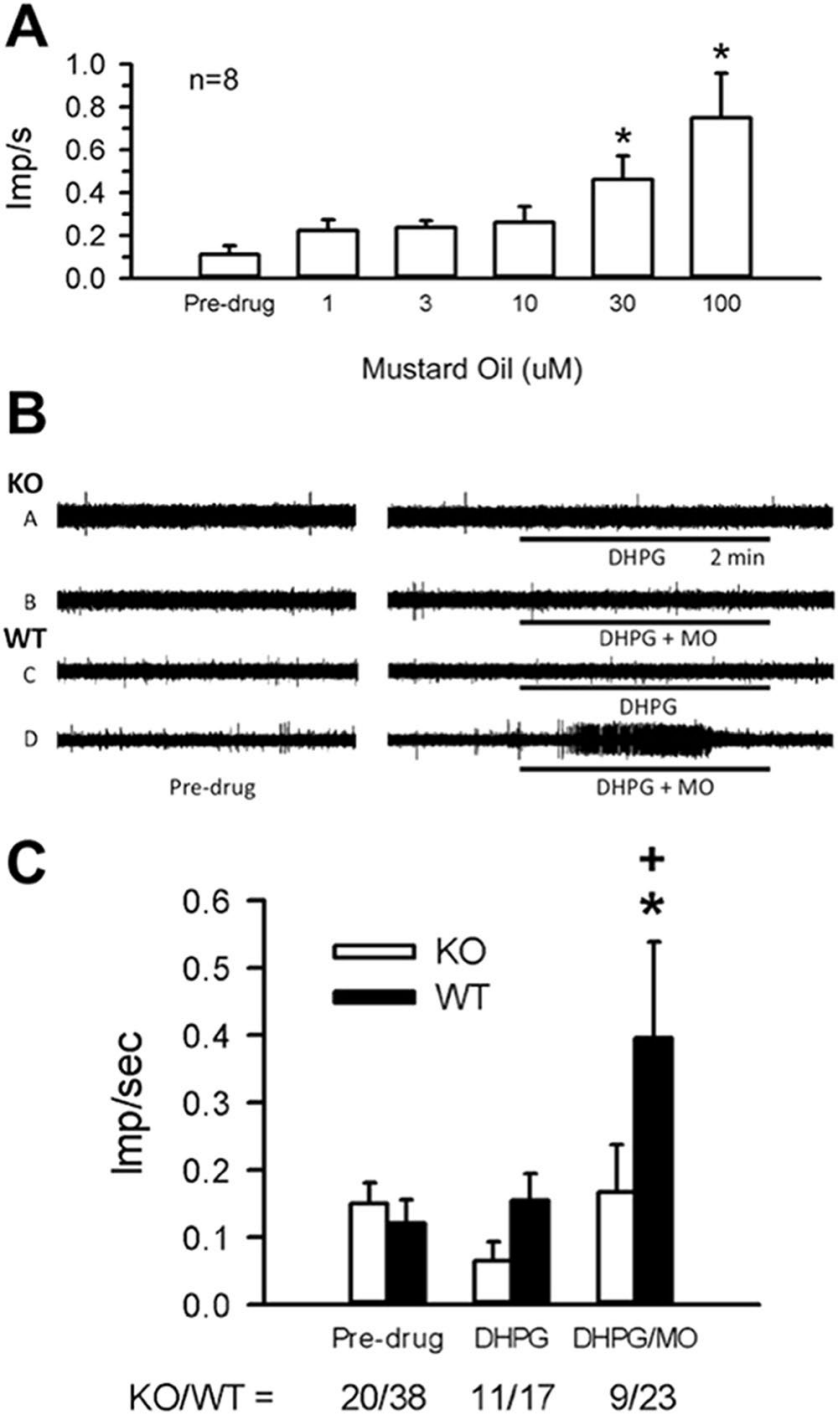
mGluR sensitization of Mechanical Responses in Nociceptors from AKAP WT and KO mice. (**A**) Discharge rates of receptive fields of WT nociceptors following mustard oil (MO) application in ascending doses (2 minutes each). *p < 0.05 vs. pre-drug baseline, Kruskal-Wallis ANOVA on Ranks with Dunn’s posthoc analysis. (**B**) Representative traces from (**A**,**B**) AKAP WT and (**C**,**D**) KO nociceptor nociceptor field activity in response to 10 µM DHPG (**A**,**C**) alone or (**B**,**D**) applied with 10 µM MO. (**C**). Collective drug-induced activity discharge rates of receptive fields from experiments conducted in panel B. *p < 0.05, significant vs. pre-drug; ^+^p < 0.05; significant vs KO at same dose; Kruskal-Wallis ANOVA on Ranks with Dunn’s posthoc analysis.

In the next series of experiments, we employed an electrophysiological approach to determine whether AKAP mediates mGluR5 sensitization of TRPA1 in sensory afferent neurons. Here, we monitored action potential generation in peripheral axons using a skin-nerve preparation approach following previously published protocols^47^. First, to determine the general degree of TRPA1-dependent nocicpetor activation, MO was applied in ascending doses (1, 3, 10, 30, 100 µM, 2 min each) to the receptive fields of nociceptors (n = 8) from WT. Exposure to MO resulted in a significant increase in the discharge rate at the 30 µM (0.46 ± 0.11 imp/sec) and 100 µM (0.75 ± 0.21 imp/sec) dose compared to pre-drug baseline (0.11 ± 0.04 imp/s, Fig. 2A). Thus, MO alone induced significant excitation of nociceptors in WT mice.

In both AKAP WT and KO mice, a 2 min exposure of nociceptor receptive fields in either KO or WT to 10 µM DHPG produced no change in nociceptor activity (WT: 0.15 ± 0.04 imp/sec; KO: 0.06 ± 0.03 imp/sec) compared to pre-drug levels (WT: 0.12 ± 0.04 imp/sec; KO: 0.15 ± 0.03 imp/sec). When 10 µM DHPG was applied in combination with 10 µM MO to WT receptive fields, a significant amount of nociceptor activity was generated (0.40 ± 0.14 imp/sec) compared to WT pre-drug levels and compared to KO (0.17 ± 0.07 imp/sec, Fig. 2B). A summary of this activity is shown in Fig. 2C. These *ex vivo* preparation findings indicate that while AKAP expression does not dictate the resting discharge rate of nociceptors, it facilitates mGluR5 sensitization of MO-responsive nociceptors.

**Figure 3:**
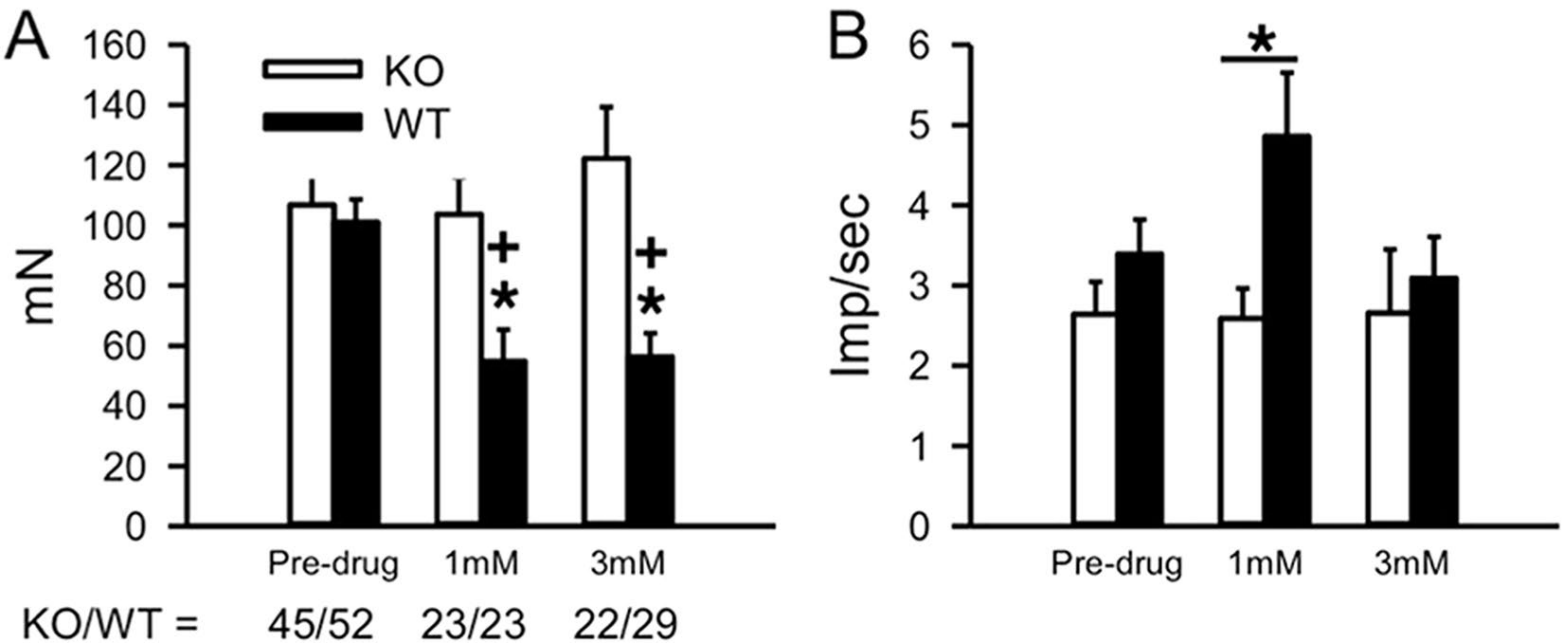
mGluR sensitization of Mustard Oil Responses in Nociceptors from AKAP WT and KO mice. (**A**) Recorded threshold to activation to a ramp stimulus following exposure to 1 mM or 3 mM DHPG from skin-nerve preparation nociceptor receptive fields of AKAP WT and KO mice and (**B**). Discharge rates to a suprathreshold mechanical stimulus (250 mN). *p < 0.05; significant vs. pre-drug; ^+^p < 0.05, significant vs. KO at same dose; Kruskal-Wallis ANOVA on Ranks with Dunn’s posthoc analysis.

Baseline mechanical thresholds were similar in AKAP WT (n = 3, 101 ± 7 mN) mice compared to KO littermates (n = 3, 106 ± 9 mN, Fig. 3A). Furthermore, the response to a suprathreshold mechanical stimulus (250mN for 10 sec) was not different in WT (3.39 ± 0.43 imp/sec) versus KO mice (2.64 ± 0.40 imp/sec, Fig. 3B). Following receptive field exposure to 1 or 3 mM DHPG, nociceptors from WT mice showed a significant decrease in the threshold to activation (1 mM: 54 ± 10 mN; 3 mM: 56 ± 7 mN) compared to pre-drug levels and compared to KO (1 mM: 103 ± 12 mN; 3 mM: 122 ± 17 mN, Fig. 3A). Surprisingly, the 1 mM, but not the 3 mM dose of DHPG enhanced nociceptor responses to the suprathreshold mechanical stimulus in WT mice (4.86 ± 0.79 imp/sec) compared to KO littermates (2.59 ± 0.37 imp/sec, Fig. 3B). These data demonstrate that while AKAP expression does not determine mechanical threshold or responses to suprathreshold stimulation, the presence of AKAP permits DHPG-induced sensitization to mechanical stimulation.

**Figure 4:**
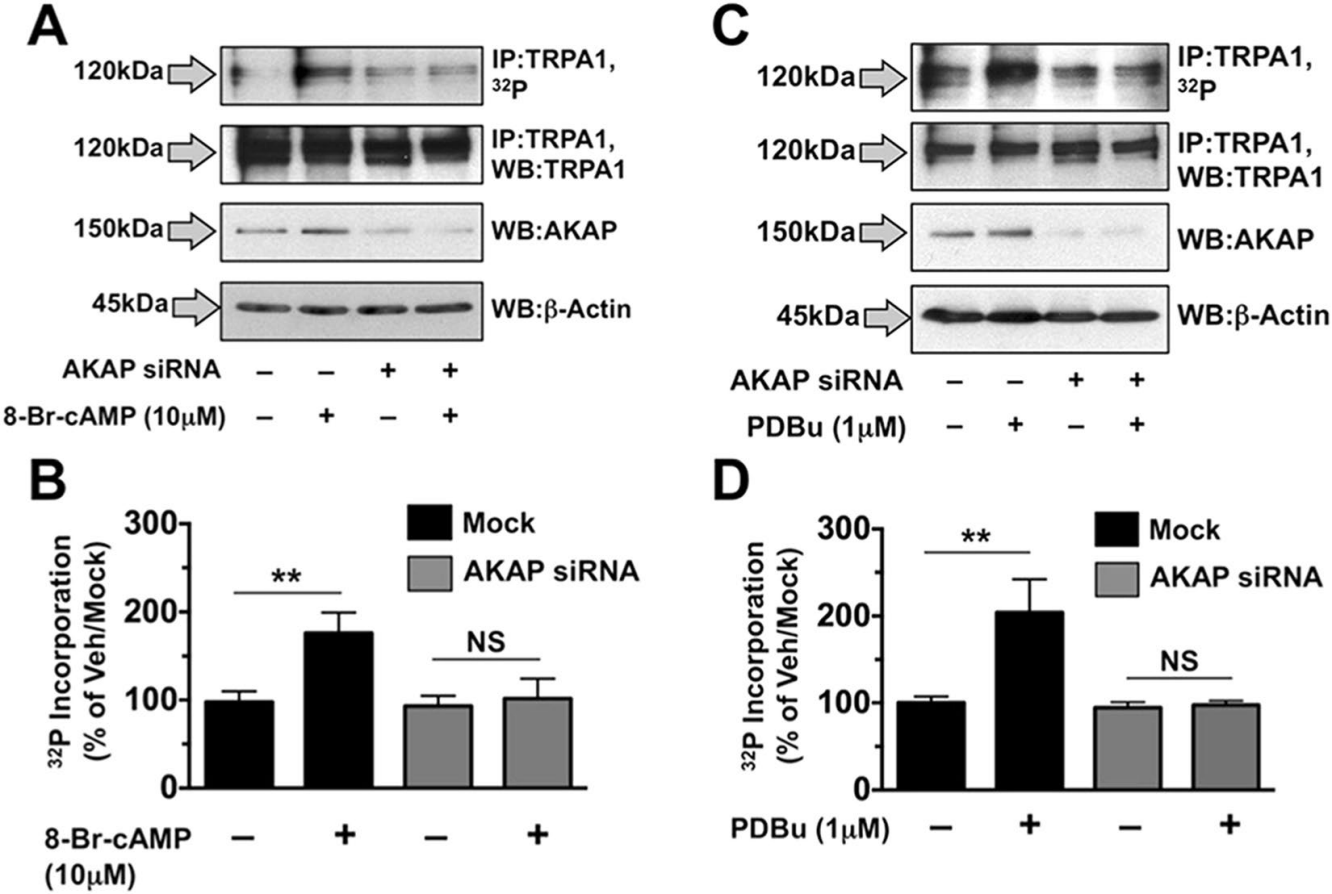
TRPA1 Phosphorylation by PKA and PKC is Dependent on AKAP Expression. Cultured rat trigeminal neurons were grown on 10-cm plates, and transfected with siRNA directed against AKAP, as illustrated. Cultures were then incubated with ^32^P-orthophosphate for 4 h, prior to treatment with vehicle 8-Br-cAMP (10 μM, **A**,**B**), or vehicle vs. phorbol 12,13-dibutyrate (PDBu, 1 μM, (**C**,**D**) for 5 min before harvesting and immunoprecipitation (IP) of TRPA1. Immunoprecipitants and aliquots of general lysates were resolved by SDS-PAGE and Western blot (WB), as depicted. Representative autoradiographic and WB results are shown (**A** and **C**). Quantified autoradiographic results were normalized to total TRPA1 IP (**B** and **D**). Results are representative of 4–5 independent trials; **p < 0.05; ns = no significance; two-way ANOVA with Bonferroni post-hoc analysis.

AKAP scaffolds enzymes including protein kinases A and C (PKA, PKC) to the plasma membrane to organize post-translational receptor modifications^1, 48^. Several labs have demonstrated a functional requirement for AKAP expression in TRPV1 phosphorylation and sensitization by PKA and PKC^3–5, 49^. However, despite reported AKAP association with TRPA1^5^, it is unknown whether TRPA1 is post-translationally modified by either PKA or PKC. In Fig. 4, we found that TRPA1 undergoes phosphorylation by both PKA and PKC in an AKAP-dependent manner. 8-Br-cAMP treatment (10 μM, 10 min) significantly increased ^32^P-orthophosphate incorporation of immunoprecipitated TRPA1 from cultured sensory neurons (Fig. 4A,C, **p ≤ 0.01, two-way ANOVA, post-hoc Bonferroni). Furthermore, siRNA-knock down of AKAP expression reduced PKA phosphorylation of TRPA1, indicating a role for the scaffolding protein in post-translational phosphorylation of TRPA1. Similarly, phorbol 12,13-dibutyrate (PDBu) treatment (PKC-activator, 1 μM, 10 min) significantly increased TRPA1 phosphorylation in an AKAP-dependent manner (Fig. 4B,D, **p ≤ 0.01, two-way ANOVA, post-hoc Bonferroni). Although previous reports identify multiple mediators of TRPA1 sensitization^9, 50, 51^, results presented here represent the first evidence of TRPA1 phosphorylation by PKA and/or PKC.

**Figure 5:**
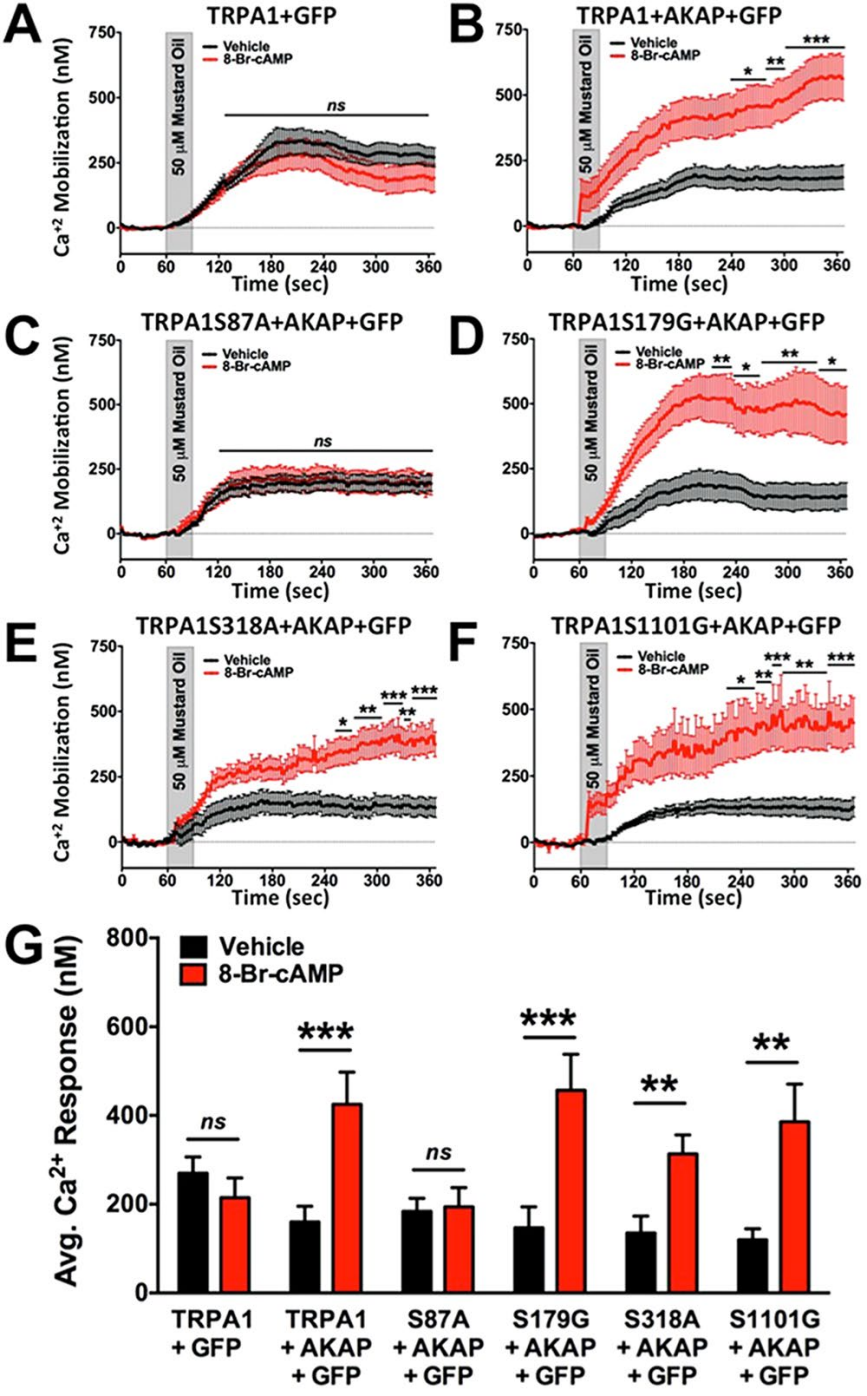
Site-Directed Mutagenesis of Potential PKA phosphorylation sites on TRPA1. Cumulative traces from Chinese hamster ovary (CHO) cells transiently transfected with various cDNAs in the following combinations: (**A**) TRPA1 and empty green fluorescence protein vector (GFP), (**B**) TRPA1, AKAP and GFP, (**C**) TRPA1 S87A, AKAP and GFP, (**D**) TRPA1 S179G, AKAP and GFP, (**E**) TRPA1 S318A, AKAP and GFP, or (**F**) TRPA1 S1101G, AKAP and GFP. Real-time Ca^2+^ accumulation measurements were recorded from GFP-positive cells following vehicle (black) or 8-Br-cAMP (10 μM, red) pre-treatment (5 min) to a mustard oil (MO, 50 μM, 30 sec) challenge. ***p < 0.005; **p < 0.01; *p < 0.05; two-way ANOVA with Bonferroni post-hoc analysis. **G**. Quantified results from cumulative recordings (n = 12–46); ns = no significance; ***p < 0.005; **p < 0.01; trace significance determined by two-way ANOVA with Bonferroni posthoc analysis and quantified data significance determined by unpaired student’s *t* test.

Next we performed site-directed mutagenesis on mouse TRPA1 cDNA to identify specific sites of phosphorylation. Reports on TRPA1 inflammatory sensitization use either human, rat, or mouse cDNA for their studies, complicating the potential for homogeneous results from individual species. In response to this, we performed CLUSTAL alignment of all three species to identify shared intracellular regions, then utilized phosphorylation site prediction algorithms (pkaPS^52^; NetPhosK1.0^53^; KinasePhos2.0^54^) to identify potential sites of PKA and/or PKC phosphorylation shared across all three species. Potential sites identified by all three algorithms were only considered if their predictive outcomes (r value) were calculated to be ≥0.75, and fell within regions of intracellular expression along the primary amino acid sequence. With this analysis, we were able to discount a large number of identified sites that were either unshared across species, did not calculate to be predictive, or exist within transmembrane or extracellular regions of the primary amino acid sequence. Four potential sites for PKA phosphorylation were identified as serine 87, serine 179, serine 318, and serine 1101, and all were mutated to either alanine or glycine amino acids to prevent phosphorylation. TRPA1 WT or mutant cDNAs were transfected into Chinese Hamster Ovary (CHO) cells along with green fluorescent protein (for positive identification of transfected cells) and either empty vector or AKAP WT cDNA. To identify which phosphorylation site(s) direct(s) PKA-dependent sensitization of TRPA1, we utilized real-time Ca^2+^ imaging to monitor MO (50 μM, 30 sec)-induced calcium accumulation in transfected CHO cells pre-incubated with vehicle or 8-Br-cAMP (10 μM, 5 min). CHO cells that co-expressed AKAP WT and TRPA1 WT resulted in significant PKA-sensitization of TRPA1, whereas co-expression of empty vector and TRPA1 WT did not (***p ≤ 0.005, unpaired student’s *t* test, Fig. 5A,B,G). Given that TRPA1 WT sensitization following PKA stimulation was dependent on AKAP expression, TRPA1 mutants were co-transfected with AKAP WT. Like TRPA1 WT, mutants S179G, S318A, and S1101G also underwent significant PKA stimulated sensitization (***p ≤ 0.005, **p ≤ 0.01, unpaired student’s *t* test, Fig. 5D–G). In contrast, mutation of TRPA1 S87 to an alanine prevented PKA-mediated sensitization of MO-induced Ca^2+^ accumulation (Fig. 5C,G). Similar basal MO response levels to CHO cells expressing WT TRPA1 and AKAP indicate that the site-mutation did not interfere with normal receptor response. Taken together, results indicate that serine 87 within the intracellular N-terminus of TRPA1 serves as a phosphorylation site for AKAP-scaffolding of PKA and mediates receptor response to agonist stimulation.

**Figure 6:**
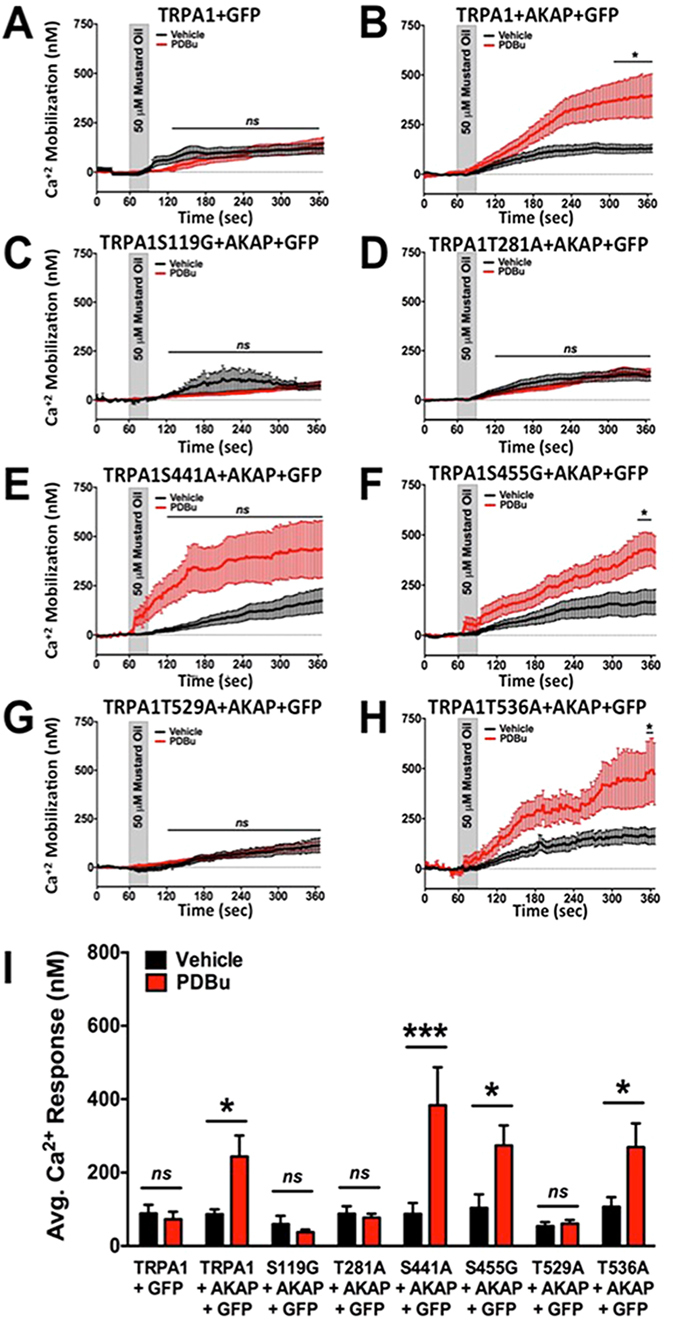
Site-Directed Mutagenesis of Potential PKC phosphorylation sites on TRPA1. Cumulative traces from Chinese hamster ovary (CHO) cells transiently transfected with various cDNAs in the following combinations: (**A**) TRPA1 and empty green fluorescence protein vector (GFP), (**B**) TRPA1, AKAP and GFP, (**C**) TRPA1 S119G, AKAP and GFP, (**D**) TRPA1 T281A, AKAP, and GFP, (**E**) TRPA1 S441A, AKAP, and GFP, (**F**) TRPA1 S455G, AKAP, and GFP, (**G**) TRPA1 T529A, AKAP, and GFP, (**H**) TRPA1 T536A, AKAP, and GFP. Real-time Ca^2+^ accumulation measurements were recorded from GFP-positive cells following vehicle (black) or PDBu (1 μM, red) pre-treatment (5 min) to a mustard oil (MO, 50 μM, 30 sec) challenge. ***p < 0.005; *p < 0.05; ns = no significance; one-way ANOVA. (**I**) Quantified results from cumulative recordings (n = 24–64/cDNA transfection paradigm), ns = no significance; ***p < 0.005; **p < 0.01; trace significance determined by two-way ANOVA with Bonferroni posthoc analysis and quantified data significance determined by unpaired student’s *t* test.

Following similar analysis, we explored potential PKC phosphorylation sites within the TRPA1 amino acid sequence. Here, CHO cells were transfected as in Fig. 6, but with predicted PKC phosphorylation sites mutated to either alanine or glycine amino acids. Six potential sites for PKC phosphorylation were identified as serine 119, threonine 281, serine 441, serine 455, threonine 529, and threonine 536. Similar to experiments in Fig. 6, AKAP, empty vector GFP and either TRPA1 WT or TRPA1 PKC phosphorylation mutants were co-transfected into CHO cells. Cells were pre-incubated in either vehicle or PDBu (1 μM, 5 min), then real-time Ca^+2^ imaging was used to measure PKC-dependent sensitization of MO (50 μM, 30 sec)-induced Ca^2+^ accumulation. PDBu-stimulated sensitization of MO-responses in WT TRPA1-transfected cells required AKAP co-expression (Fig. 6A,B,I). Additionally, site-directed mutations of TRPA1 at serine 441, serine 455 and threonine 536 had no effect on PDBu sensitization of MO-response (Fig. 6E,F,H,I). In contrast, mutations of serine 119, threonine 281, and threonine 529 to non-phosphorylatable amino acid residues ablated any PKC-directed sensitization in MO response (Fig. 6C,D,G,I). Basal MO-induced Ca^2+^ accumulation amounts were similar across vehicle-treated cells that expressed these mutants. Therefore, results indicate that AKAP is required for PKC-sensitization of TRPA1, and that S119, T281, and T529 residues exist as PKC-modifiable amino acid residues.

**Figure 7:**
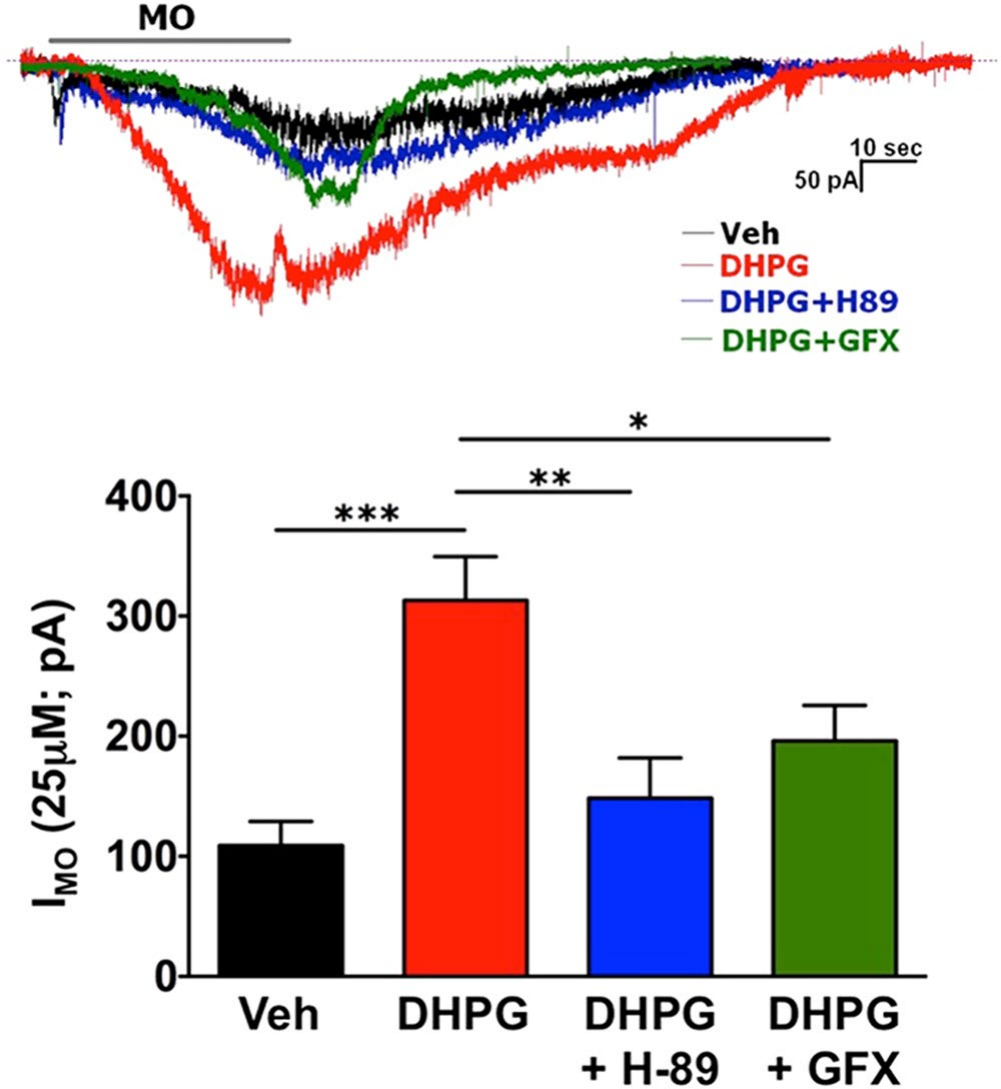
mGluR5-sensitization of TRPA1 Current is Sensitive to PKA and PKC Inhibition. Mustard oil (MO; 25 μM)-activated current was recorded from DRG sensory neurons. MO was applied for 30 sec. Vehicle (ddH2O), DHPG, H89 (PKA inhibitor) and GFX (PKC inhibitor) were applied at concentrations of 10 μM (each). DRG neurons were incubated for 10 min with drugs marked on X-axis, followed by co-application of MO. *p < 0.05; **p < 0.01; ***p < 0.001; n = 7–10 MO responsive cells; one-way ANOVA with Bonferroni’s post-hoc analysis.

Since TRPA1 is modified by PKA and PKC (Figs 4–6), we next sought to determine the sensitivity of DHPG-sensitization of MO-current to inhibition of the two kinases. For this experiment, we employed patch-clamp electrophysiology of isolated DRG sensory neurons following serum-starvation for 18 h. MO (25 μM, 30 s) currents from DRG neurons pre-treated with mGluR5 agonist DHPG (10 μM, 60 s) increased 3-fold over vehicle treated neurons (Fig. 7, ***p ≤ 0.005, one-way ANOVA with post hoc Bonferroni analysis). However, in DRG neurons pre-inubated with PKA inhibitor H-89 (10 μM) or PKC inhibitor GF 109203X (GFX, 10 μM), DHPG-sensitized MO-currents were reversed, indicating that mGluR5 sensitization of TRPA1 requires functional PKA and PKC activity.

**Figure 8:**
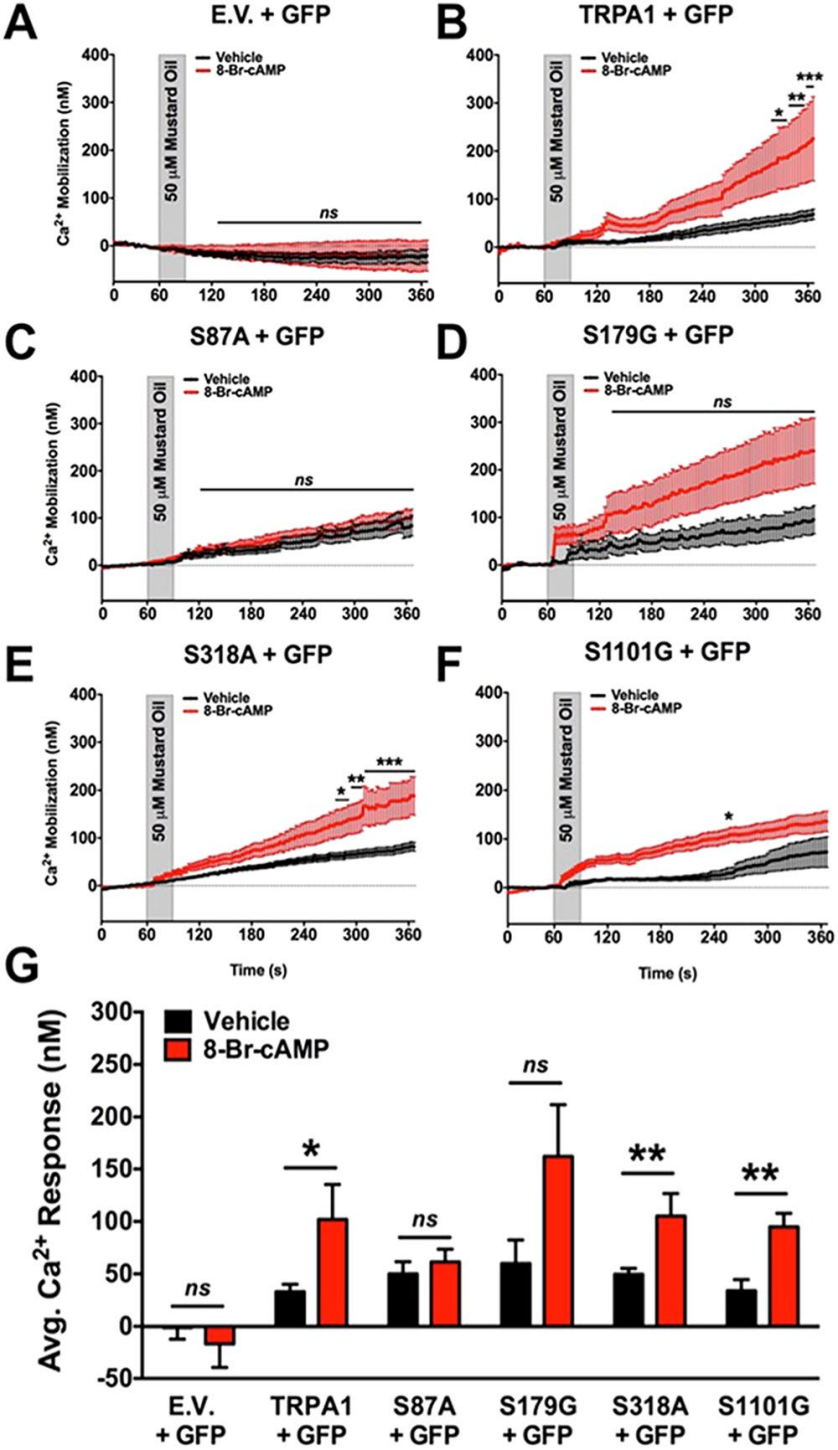
8-Br-cAMP-sensitization of TRPA1 Requires PKA-phosphorylation Site. Mustard oil (MO, 50 μM)-activated Ca^2+^ mobilization was monitored from DRG sensory neurons harvested from TRPA1 KO mice and transfected with cDNA corresponding to green fluorescent protein (eGFP-N1, GFP) and either pcDNA3 (empty vector, E.V., **A**), mouse TRPA1 WT (TRPA1, **B**), mouse TRPA1 S87A (S87A, **C**), mouse TRPA1 S179G (S179G, **D**), mouse TRPA1 S318A (S318A, **E**), or mouse TRPA1 S1101G (S1101G, **F**). For 5 min prior to recordings, DRG were pre-incubated for 5 min in vehicle (ddH2O) or 8-Br-cAMP (10 μM), then DRG were challenged with MO (30 sec) and Ca^2+^ accumulation was measured. Cumulative traces are displayed in panels **A**–**F**, quantitative measurements from multiple neurons displayed in panel **G**. *p < 0.05; **p < 0.01; ns = no significance; n = 15–39 MO responsive cells per TRPA1 cDNA transfection; trace significance determined by two-way ANOVA with Bonferroni posthoc analysis and quantified data significance determined by unpaired student’s *t* test.

**Figure 9:**
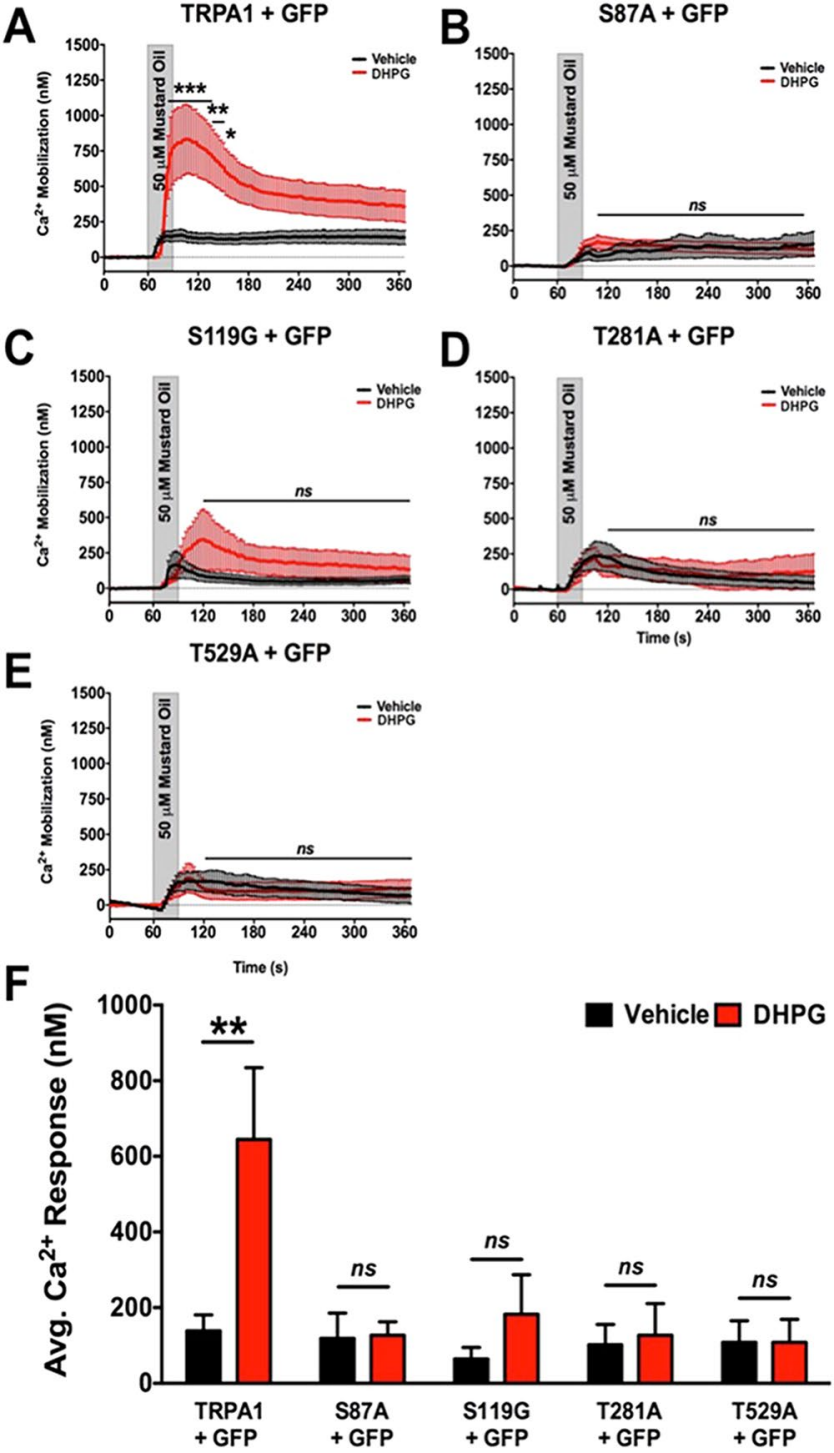
mGluR5-sensitization of TRPA1 Requires PKA and PKC-phosphorylation Sites. Mustard oil (MO, 50 μM)-activated Ca^2+^ mobilization was monitored from DRG sensory neurons harvested from TRPA1 KO mice and transfected with cDNA corresponding to green fluorescent protein (eGFP-N1, GFP) and either mouse TRPA1 WT (TRPA1, **A**), mouse TRPA1 S87A (S87A, **B**), mouse TRPA1 S119G (S119G, **C**), mouse TRPA1 T281A (T281A, **D**), or mouse TRPA1 T529A (T529A, **E**). For 5 min prior to recordings, DRG were pre-incubated for 5 min in vehicle (ddH2O) or DHPG (10 μM), then DRG were challenged with MO (30 sec) and Ca^2+^ accumulation was measured. Cumulative traces are displayed in panels **A**–**E**, quantitative measurements from multiple neurons displayed in panel **F**. **p < 0.01; ns = no significance; n = 21–35 MO responsive cells per TRPA1 cDNA transfection; trace significance determined by two-way ANOVA with Bonferroni posthoc analysis and quantified data significance determined by unpaired student’s *t* test.

Lastly, we examined whether mutating PKA and/or PKC phosphorylation sites on TRPA1 would affect sensitization of MO responses in nucleofected mouse DRG neurons. Neurons were cultured from L4-L6 DRG of TRPA1 KO mice, and nucleofected with cDNAs corresponding to mouse TRPA1 WT, S87A, S179G, S318A, or S1101G, along with GFP empty vector for identification of positively nucleofected neurons. Prior to MO (50 μM, 30 sec) stimulation, DRG cultures were pre-incubated with 8-Br-cAMP (10 μM, 5 min) to activate PKA-dependent phosphorylation processes. Experimental results revealed that S87 in the N-terminus of TRPA1 is important to PKA-mediated sensitization of an MO response in DRG neurons (Fig. 8). These results support that a sensitizing PKA phosphorylation site exists on mouse TRPA1. To build on this, we similarly cultured DRG neurons from TRPA1 KO mice and nucleofected them with wild-type and mutated TRPA1 cDNA in which PKA- and PKC-sensitive sites are mutated. Next, nucleofected DRG cultures were pre-incubated with mGluR5 agonist DHPG (10 μM, 5 min) to determine amino acid requirements for TRPA1 sensitization. In Fig. 9, observed results indicate that all of the amino acid sites, including S87, S119, T281, and T529, are important to mGluR5-sensitization of TRPA1 responses in cultured DRG neurons. Taken together, data presented herein identify important sites that modulate PKA- and PKC-sensitization of TRPA1, and play a pivotal role in mGluR5 sensitization of TRPA1 in DRG neurons.

## Discussion

Multiple changes throughout the peripheral and central nervous system contribute to the transition from acute to chronic pain. While many have documented changes in secondary afferent neuronal sensitivities in correlation with chronic pain, few have explored biochemical changes that occur with the primary afferent fiber. Here, we have investigated how the inflammatory mediator glutamate sensitizes pain-sensing neurons to mechanical stimuli along a persistent timeline through the AKAP-dependent, post-translational modification of TRPA1. This finding represents an innovative benchmark for studying future modifications of TRPA1 that may also contribute to increased nociceptive sensitivities associated with chronic pain syndromes.

Group 1 metabotropic glutamate receptors such as mGluR5 are expressed by primary afferent neurons and are activated by the inflammatory mediator glutamate, promoting neurogenic inflammation. Gαq/11-PLC signaling pathway association with mGluR5 would indicate that downstream effectors could contribute to persistent pain symptoms such as mechanical hypersensitivity. Hyperalgesic priming behavior measures not only support the contribution of mGluR5 to pain “chronification”, but also indicate roles for AKAP and TRPA1. Importantly, these are the first reported results indicating a role for the scaffolding protein AKAP and the ligand-gated ion channel TRPA1 in persistent mechanical hypersensitivity. Additional *ex vivo* electrophysiology confirmed the importance of AKAP in mGluR5 sensitization of mechanical responses and MO application. Together, these findings indicate that AKAP plays a role in regulating the post-translational modification of TRPA1.

Given the ubiquitous anatomical distribution of AKAP in primary afferent neurons, co-expression with TRPA1 would suggest an accessibility of the scaffolding protein to mediate TRPA1, perhaps via phosphorylation. Indeed, radioactive labeling results indicate that TRPA1 is phosphorylated by PKA and PKC in primary afferent cultures. Additional site-directed mutagenesis confirmed specific sites of TRPA1 post-translational modification in an AKAP-dependent manner. Furthermore, mGluR5 sensitization of TRPA1 was blocked by PKA- or PKC-inhibition, and was ablated in DRG neurons cultured from TRPA1 KO mice nucleofected with TRPA1 mutants in which PKA- and/or PKC-specific phosphorylation sites were mutated. Together, these findings indicate a likely involvement of AKAP scaffolding of kinases in the mechanical hypersensitization associated with chronic pain through post-translational phosphorylation of TRPA1 in primary afferent nociceptors.

Studies over the past decade have identified TRPA1 as a key component in the mechanical firing of nociceptors. TRPA1 expression is required for mechanical current generation in cutaneous sensory neurons^22, 24, 55, 56^, including during inflammation^43^. These studies are crucial considering the number of reports that incriminate TRPA1 in pathological mechanical pain conditions including persistent craniofacial pain^57^ and arthritis^42, 58–60^. Furthermore, TRPA1 has been reported to play a significant role in respiratory inflammation^61^ and contact dermatitis^62^. These pathologies, all of which have been known to exist in chronic states, highlight the importance of TRPA1 transduction, and the inflammatory role that the ligand-gated ion channel plays.

Inflammation drives post-translational modification of a number of biochemical targets expressed in nociceptors, including TRP channels such as TRPV1. Previous work from multiple research groups demonstrate TRPV1 phosphorylation by PKC and PKA following exposure to various inflammatory mediators^63–66^, even working through Type 1 glutamate receptors^67^, thereby reducing the threshold for channel activation^68^ and increasing the likelihood for nociceptor depolarization^69^. Following this work, AKAP was discovered to scaffold multiple signaling events in nociceptors, including PKC and PKA phosphorylation of TRPV1 following exposure to inflammatory mediators^3–5, 49^. Previous reports on the efficacy of mGluR5 agonists on nociceptor sensitization^11^ led to the discovery that AKAP serves to mediate mGluR5 sensitization of TRPV1 and contributes to persistent thermal hypersensitivity in a hyperalgesic priming model^37^. Additional investigations reported herein serve as the next logical step in determining important biochemical players that contribute to persistent mechanical hypersensitivity reported as a symptom in multiple chronic pain syndromes.

The lack of prior TRPA1 phosphorylation studies is understood for several reasons. Firstly, the Neuromics antibody used to target rat TRPA1 in these studies (see Materials and Methods) was first made available just several years ago, proving to be highly specific for both immunoprecipitation and Western blot analysis. Second, the CLUSTAL alignment of human, mouse, and rat sequences to determine homology of highest percentage sites for possible PKC and/or PKA phosphorylation was likely not used previously. This approach is not only more physiologically relevant, by maintaining genetic homology across species, but reduces the likelihood for negative outcomes by chasing unimportant amino acid targets. Thirdly, previous studies that have characterized TRPA1 sensitization were conducted in immortalized cell systems^70^, such that transfected TRPA1 receptor channels over express compared to endogenous-to-no expression of scaffolding proteins such as AKAP. Such model systems would prevent PKA- and/or PKC-mediated sensitization, provided the lack of equally-expressed AKAP, as afforded by results demonstrated here in. Indeed, TRPA1 sensitization in more physiologically-relevant cell models demonstrate sensitivity to protein kinase manipulation^71, 72^.

TRPA1 biochemical sensitization has previously been described as a process that occurs through cysteine manipulations in the intracellular N-terminal amino acid sequence^73, 74^. Indeed, several groups have reported that covalent modifications to these N-terminal cysteines are capable of activating TRPA1^23, 25^. However, inflammatory sensitization has also been reported for TRPA1^9, 75^, with little mechanistic explanation provided. Here, we identify a post-translational, phosphorylation mechanism for inflammatory sensitization of TRPA1, as well as individual sites of modification. It is possible that phosphorylation events support or are supported by covalent cysteine modifications, in light of increased knowledge concerning allosteric remodeling of ligand-gated ion channels^76^, including TRPA1^77^. It is doubtful that either of these post-translational events occurs in singularity. Furthermore, it is likely that TRPA1 phosphorylation contributes to other painful pathologies associated with the ligand-gated ion channel, including chemotherapy-induced peripheral neuropathy (CIPN^50, 78^) and/or nerve crush/ligation^79^). However, the discovery of TRPA1 phosphorylation and its impact towards persistent mechanical hypersensitivity provides a mechanism to target in the search for new analgesic compounds.

## Electronic supplementary material

Supplementary Information
