# Supplementary material for: A-Kinase Anchoring Protein 79/150 Scaffolds Transient Receptor Potential A 1 Phosphorylation and Sensitization by Metabotropic Glutamate Receptor Activation
Source: Sci Rep. 2017 May 12;7:1842. doi: 10.1038/s41598-017-01999-4 (PMC5431798; doi:10.1038/s41598-017-01999-4)
Supplement: Supplementary file 1 — Supplementary Information [file 41598_2017_1999_MOESM1_ESM.pdf]

A-Kinase Anchoring Protein 79/150 Scaffolds Transient Receptor Potential A 1 Phosphorylation and Sensitization by Metabotropic Glutamate Receptor Activation

Allison Doyle Brackley[1], Ruben Gomez[2], Kristi A. Guerrero[2], Armen N. Akopian[3], Marc J. Glucksman[4], Junhui Du[4], Susan M. Carlton[5], Nathaniel A. Jeske[1, 2, 6]

Supplementary Figure 1

1	MKRSLRRVLR	PEERKEVQGV	VYRGVGKDMD	CSKESFKVDI	EGDMCRLEAF
51	IKNRRKLSKY	EDENLCLLHH	AAAEGQVELM	QLIINGSSCE	ALNVMDDYGN
101	TPLHWAAEKN	QVESVKFLLS	QGANPNLRNR	NMMAPLHIAV	QGMYNEVIKV
151	LTEHKATNIN	LEGENGNTAL	MSTCAKDNSE	ALQILLEKGA	KLCKSNKWGD
201	YPVHQAAFSG	AKRCMELILA	YGEKTGYSRE	AHINFVNHKK	ASPLHLAVQS
251	GDLDMIKMCL	DSGAHIDMME	NAKCMALHFA	ATQGATDIVK	LMISSYTGSS
301	DIVNAVDGNQ	ETLLHRASLF	DHHDLADYLI	SVGADINSTD	SEGRSPLILA
351	TASASWNIVN	LLLSKGAKVD	IKDHLGRNFL	HLTVQQPYGL	RNLRPEFLQM
401	QHIKELVMDE	DNDGCTPLHY	ACRQGAPVSV	NNLLRFNVSV	HSKSKDKKSP
451	LHFAASYGRI	NTCQRLLQDI	SDTRLLNEGD	LHGMTPLHLA	AKNGHDKVVQ
501	LLLKKGALFL	SDHNGWTALH	HASMGGYTQT	MKVILDTNLK	CTDRLDEEGN
551	TALHFAAREG	HAKAVAMLLS	YNADILLNKK	QASFLHIALH	NKRKEVVLTT
601	IRSKRWDECL	QVFTHDSPSN	RCPIMEMVEY	LPECMKVLLD	FCMIPSTEDK
651	SCQDYHIEYN	FKYLQCPLSM	TKKVTPTQDV	IYEPLTILNV	MVQHNRIELL
701	NHPVCREYLL	MKWCAYGFRA	HMMNLGSYCL	GLIPMTLLVV	KIQPGMAFNS
751	TGIINETIST	HEERINTLNS	FPLKICMILV	FLSSIFGYCK	EVVQIFQQKR
801	NYFLDYNNAL	EWVIYTTSMI	FVLPLFLDIP	AYMQWQCGAI	AIFFYWMNFL
851	LYLQRFENCG	IFIVMLEVIF	KTLLRSTGVF	IFLLLAFGLS	FYVLLNFQDA
901	FSTPLLSLIQ	TFSMMLGDIN	YRDAFLEPLF	RNELAYPVLT	FGQLIAFTMF
951	VPIVLMNLLI	GLAVGDIAEV	QKHASLKRIA	MQVELHTNLE	KKLPFWYLRK
1001	VDQRSTIVYP	NRPRHGRMLR	FFHYFLSMQE	TRQEAPNIDT	CLEMEILKQK
1051	YRLKDLTSLL	EKQHELIKLI	IQKMEIISET	EDEDNHCSFQ	DRFKKERLEQ
1101	MHSKWNFVLN	AVKTKTHCSI	SHPDI		

Supplementary Figure 1. TRPA1 Antibody Verification. Rat TRPA1 sequence is indicated, with thirteen tryptic fragments sequenced by mass spectroscopic analyses (in yellow) of immunoprecipitated TRPA1.
